# Epidemiological and etiological characteristics of mild hand, foot and mouth disease in children under 7 years old, Nanjing, China, 2010–2019

**DOI:** 10.1186/s13690-022-00974-4

**Published:** 2022-10-08

**Authors:** Junjun Wang, Songning Ding, Weijia Xie, Taiwu Wang, Ying Qin, Jiandong Zheng, Xiaokun Yang, Hongting Zhao, Zhibin Peng, Tao Ma

**Affiliations:** 1 Nanjing Municipal Center for Disease Control and Prevention, No. 2 Zizhulin, Nanjing, 210003 Jiangsu China; 2 Division of Infectious Disease, Key Laboratory of Surveillance and Early-Warning On Infectious Disease, Chinese Center for Disease Control and Prevention, 155 Changbai Road, Changping District, Beijing, 102206 China; 3 Chinese Field Epidemiology Training Program, Chinese Center for Disease Control and Prevention, Beijing, China; 4 Department of Acute Infectious Diseases Control and Prevention, Nanjing Municipal Center for Disease Control and Prevention, No. 2 Zizhulin, Nanjing, 210003 Jiangsu China; 5 Department of Epidemiology, College of Preventive Medicine, Army Medical University, Chongqing, China; 6 Center for Disease Control and Prevention of Eastern Theatre Command, Nanjing, Jiangsu China

**Keywords:** Hand, foot and mouth disease, Mild cases, Epidemiology, Coxsackievirus A16, Coxsackievirus A6, FleXScan

## Abstract

**Background:**

Mild hand, foot and mouth disease (HFMD) cases make up a relatively high proportion of HFMD while have often been overlooked. This study aimed to investigate the epidemiological and etiological characteristics of mild HFMD in Nanjing.

**Methods:**

Data on mild HFMD cases, during 2010–2019 in Nanjing, were collected from the China Information System for Disease Control and Prevention. This study mainly focused on mild cases aged < 7 years. Descriptive analysis was used to summarize epidemiological and etiological characteristics of mild cases. Flexible spatial scan statistic was used to detect spatial clusters of mild cases.

**Results:**

A total of 175,339 mild cases aged < 7 years were reported, accounting for 94.4% of all mild cases. There was a higher average annual incidence of mild HFMD in children aged < 7 years (4,428 cases/100,000) compared with children aged ≥ 7 years (14 cases/100,000, *P* < 0.001), and especially children aged 1-year-old (7,908 cases/100,000). Mild cases showed semi-annual peaks of activity, including a major peak (April to July) and a minor peak (September to November). The average annual incidence was higher in males (5,040 cases/100,000) than females (3,755 cases/100,000). Based on the cumulative reported cases, the most likely cluster was detected, including Yuhuatai District, Jiangning District, Jiangbei new Area, and Pukou District. The annual distribution of enterovirus serotypes showed a significant difference. During 2010–2016, Enterovirus 71 (EV71), Coxsackievirus A16 (Cox A16), and other non-EV71/Cox A16 EVs, accounted for 29.1%, 34.6%, 36.3% of all the enterovirus test positive cases, respectively. Moreover, during 2017–2019, Cox A6, Cox A16, EV71, and other non-EV71/Cox A16/Cox A6 EVs, accounted for 47.3%, 32.5%, 10.7%, 9.5%, respectively.

**Conclusions:**

Children under 7 years old are at higher risk of mild HFMD. Regions with high risk are mainly concentrated in the areas surrounding central urban areas. Cox A16 and Cox A6 became the dominant serotypes and they alternated or were co-epidemic. Our findings could provide valuable information for improving the regional surveillance, prevention and control strategies of HFMD.

**Supplementary Information:**

The online version contains supplementary material available at 10.1186/s13690-022-00974-4.

## Findings and recommendations for public health

- Mild cases of hand, foot and mouth disease (HFMD) accounted for more than 90% of HFMD cases in Nanjing over the past 10 years. Assessment, monitoring, and surveillance of mild HFMD should be strengthened.
- Mild HFMD primarily affects children aged < 7 years, especially 1-year-old. Health education and preventive measures training about HFMD should be provided and strengthened for staffs of childcare facilities, guardians, and parents of younger children.
- The high-risk regions of mild HFMD are mainly concentrated in the areas surrounding central urban areas, where weak points of HFMD control are urgently needed to be identified and improved.
- Cox A6 emerged as a new predominate serotype of mild HFMD. It is necessary to involve more other enterovirus serotypes in routine serotypes surveillance.

## Background

Hand, foot and mouth disease (HFMD) is one of the most common infectious diseases among children, which has been reported in most regions around the world [1]. HFMD seriously threatens the health of children because of its high transmissibility, complicated transmission route, and high epidemic intensity. In some Asian countries, millions of HFMD patients have caused 96,900 age-weighted disability-adjusted life-years (DALYs) per annum [2]. In 2019, the number of HFMD cases was 1.91 million, which was the second highest number of notifiable infectious diseases in mainland China [3].

The causative agents of HFMD are associated with the *Enterovirus (EV) A species* in the Enterovirus genus [4], which consists of 25 serotypes at present [5]. The main serotypes that cause HFMD are Enterovirus71 (EV71) and Coxsackievirus A16 (Cox A16) [6]. In recent years, the number of other serotypes of *EV A species* isolated from HFMD patients is increasing, such as Coxsackievirus A6 (Cox A6) and Coxsackievirus A10 (Cox A10) [4]. EV71 vaccine could provide protection for severe HFMD cases (hereinafter referred to as severe cases) infected by EV71 serotype, but provided no cross-protection against Cox A16 and other enterovirus serotypes, which might influence the distribution of the enterovirus serotypes [7].

Mild HFMD cases (hereinafter referred to as mild cases) are mainly caused by Cox A16 and other enterovirus serotypes [7]. Previous studies mainly focused on severe cases. However, most patients with enterovirus infection were mild or asymptomatic, who are major contributors to HFMD transmission [8]. Nanjing is the capital city of Jiangsu Province, located in eastern China with high HFMD incidence [9]. The number of severe cases reduced after the introduction of the EV71 vaccine in Nanjing. However, the number of the overall HFMD cases showed no significant reduction, and still ranked at the top of notifiable infectious diseases reported in Nanjing in recent years. Mild cases become a significant challenge for HFMD control. Less is known regarding the epidemic trends of mild cases, especially the change of pathogenic spectrum after the EV71 vaccination program.

In recent years, most mild cases were aged under 7 years old in Nanjing. In this study, we based on long-term surveillance data to explore the epidemic trend of mild HFMD in Nanjing, focusing on the epidemiological and etiological characteristics of the high risk group (mild cases aged < 7 years). We aimed to identify populations at higher risk, high risk areas, and circulating enterovirus serotypes to improve the surveillance and shape the targeted prevention and control measures of HFMD.

## Methods

### Data sources

We collected data on HFMD cases from the “infectious disease surveillance system” of the China Information System for Disease Control and Prevention (CISDCP). We selected the onset date from 1st January 2010 to 31 December 2019 and the current address belongs to “Nanjing municipal city”. Data of HFMD cases mainly included demographic information (e.g. gender, date of birth, and address), clinical information (e.g. dates of symptom onset and diagnosis, case type including clinical case and laboratory-confirmed case), and laboratory testing results. Annual population data for the years 2010–2019 were also collected from the “basic information system” of the CISDCP.

### Case definition

The diagnosis of HFMD case was based on the “*guidelines for the diagnosis and treatment of hand, foot and mouth disease* (2010 edition)” and “*guidelines for the diagnosis and treatment of hand, foot and mouth disease* (2018 edition)” developed by National Health Commission of China (formerly named the Ministry of Health of China) [6, 10]. A clinical case was defined as a patient who had fever, papulovesicular rash on hands, feet, oral cavity, and buttocks, with or without symptoms such as cough, rhinorrhea, anorexia, and other non-specific systemic symptoms [6, 10]. A laboratory-confirmed case was defined as a clinical case with confirmed virological evidence, including detection of specific enterovirus nucleic acid sequences (including EV71, Cox A16, and other enterovirus serotypes), or isolation and identification enteroviruses such as Cox A16, EV71 or other enterovirus serotypes that could cause HFMD [6, 10]. A clinical or laboratory-confirmed case was classified as a severe or mild case by clinicians, considering whether there were any related complications, such as nervous system complications (aseptic meningitis, encephalitis, encephalomyelitis, acute dyskinesia or autonomic nervous system dysregulation), and/or cardiorespiratory complications (pulmonary edema, pulmonary hemorrhage, or cardiopulmonary failure) [6, 11]. Once a case had been diagnosed as a clinical or laboratory-confirmed case, the medical institution must upload standardized data of the case to the CISDCP within 24 h [6, 10].

### Enterovirus detection and serotyping

For a routine enterovirus serotypes surveillance, medical personnel or staff of Center for Disease Control and Prevention (CDC) collected specimens (throat or rectal swab) and transferred them to the local CDC laboratory for enterovirus nucleic acid test by real-time reverse transcription-polymerase chain reaction (RT-PCR) or fluorescence quantitative RT-PCR. Upon a specimen with a positive enterovirus test, it will be further examined for specific enterovirus serotypes including EV71, Cox A16, and Cox A6 (Cox A6 was added to the routine serotype detection since 2017). The test results were uploaded to the CISDCP and classified into 5 categories, including EV negative, EV71 positive, Cox A16 positive, Cox A6 positive, and other EVs (non-EV71/Cox A16, or non-EV71/Cox A16/Cox A6) positive without further serotyping [6]. To reflect the changes in circulating enterovirus serotypes spectrum, analysis of the enterovirus serotypes distribution was classified into two periods before and after the implementation of Cox A6 detection.

### Statistical and geographic analysis

Descriptive statistics were conducted with SPSS 12.0. Chi-square (χ^2^) test was used to compare categorical variables. All statistical tests were two-sided, and a *p*-value < 0.05 was considered statistically significant.

Tango’s flexible spatial scan statistic was conducted along with FLeXScan 3.1.2 [12, 13]. Based on the observed number of cases and the null expected number of cases within and outside the scan window, the restricted log likelihood ratio (LLR), the test statistic, was computed under the Poisson assumption [14]. The value of parameter alpha for restriction LLR was set at 0.2 as the default in FleXScan [14]. The *p*-value of the test statistic was calculated using 999 replications for the Monte Carlo simulations. Significance level *α* was set at 0.05. The set of potential cluster regions that attained the maximum log LLR was defined as the most likely cluster (MLC), while for the other regions with scanning window of log LLR value still showing statistical significance was defined as the secondary cluster [15]. Overall relative risk (RR) was estimated for the detected cluster. All results were visualized with ArcGIS 10.3.

## Results

### Incidence feature

A total of 185,717 mild cases (including clinical cases and laboratory-confirmed cases) were reported in Nanjing from 2010 to 2019, with an average annual incidence of 231 cases/100,000 (range from 168 cases/100,000 to 356 cases/100,000). The majority of mild cases (94.4%; *n* = 175,339) were cases aged < 7 years. The annual proportion of mild cases aged < 7 years were all more than 90% among mild cases each year from 2010–2019. Moreover, children aged < 7 years had significant higher average annual incidence of mild HFMD (4,428 cases/100,000; range from 2,510 cases/100,000 to 7,220 cases/100,000) compared with children aged ≥ 7 years (14 cases/100,000; range from 7 cases/100,000 to 28 cases/100,000) (*P* < 0.001) (see Table 1 and Supplementary results). Thus, results from this study focused on mild cases aged < 7 years. For mild cases aged < 7 years, annual incidence increased yearly during 2010–2014 and increased every other year during 2014–2019 (Table 1).

**Table 1:** The annual numbers and incidence rate of mild hand, foot and mouth disease cases aged < 7 years reported in Nanjing, China, 2010–2019

**Year**	**Number of mild HFMD cases**^a^	**Mild HFMD cases aged < 7 years**		
		**Number of cases**	**Incidence (per 100,000 populations)**^b^	**Percentage (%)**^c^
2010	12,537	12,046	2510	96.1
2011	15,369	14,829	3657	96.5
2012	16,775	16,085	4237	95.9
2013	16,395	15,762	4293	96.1
2014	24,617	23,338	6278	94.8
2015	16,663	15,785	4235	94.7
2016	24,757	23,282	6236	94.0
2017	13,911	12,989	3554	93.4
2018	29,701	27,499	7220	92.6
2019	14,992	13,724	2961	91.5
Total	185,717	175,339	4428	94.4

*HFMD* Hand, foot and mouth disease

^a^Number of reported mild HFMD cases

^b^The denominator of incidence rate of reported mild HFMD cases aged < 7 years was the number of populations aged < 7 years in Nanjing

^c^Percentage showed the proportion of mild cases aged < 7 years in the reported mild cases across all age groups each year

### Seasonal distribution

**Fig. 1:**
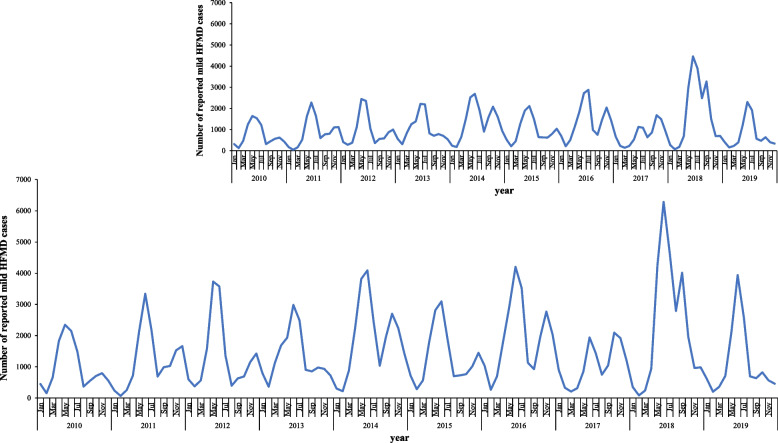
Mild HFMD cases aged < 7 years reported monthly in Nanjing, China, 2010–2019 (Distribution on the top right corner based on mild hand, foot and mouth disease (HFMD) cases aged 0–3 years)

Mild cases were reported each month, and showed semi-annual peaks, including a major peak and a smaller peak each year. A major peak occurred from April to July (spring and early summer), accounting for 57.13% (100,165/175,339) of the total number of reported cases in the whole year. A smaller peak occurred from September to November (autumn), accounting for 22.75% (39,885/175,339) of the total number of reported cases in the whole year. In contrast, February and August were the low points of the number of reported cases. The seasonal pattern of cases among preschoolers aged 0–3 years was the same as that of children aged < 7 years (Fig. 1).

### Age and gender distribution

From 2010 to 2019, the average annual incidence of mild HFMD in the 7 age groups ranged from high to low was in 1-year-old (7,908 cases/100,000), 3 years old (6,661 cases/100,000), 2 years old (6,121 cases/100,000), 4 years old (4,331 cases/100,000), 5 years old (2,486 cases/100,000), 12 months below (2,103 cases/100,000), and 6 years old (1,223 cases/100,000), which was significantly different among age groups (χ^2^ = 5233.82, *P* < 0.001). The highest average annual incidence of mild HFMD was in 1-year age group for children aged < 7 years. Moreover, the average annual incidence of males (5,040 cases/100,000) was significantly higher than females (3,755 cases/100,000) (χ^2^ = 385.80, *P* < 0.001), and the incidence of males in each year from 2010–2019 were all higher than females (Table 2).

**Table 2:** Annual incidence rates (per 100,000 population) of mild hand, foot and mouth disease among children aged < 7 years by age group and gender in Nanjing, China, 2010–2019

**Demographics**	**2010**	**2011**	**2012**	**2013**	**2014**	**2015**	**2016**	**2017**	**2018**	**2019**	**Total**
**Age**											
< 12 months	1305	1452	1699	3003	2641	2330	2730	2161	4484	746	2103
1 year old	3650	5497	6497	9525	11,059	7991	11,704	7025	16,500	3468	7908
2 years old	4200	5475	5873	5734	8670	5659	7877	4205	9269	4677	6121
3 years old	4120	6683	6751	5525	9338	6139	9061	5083	9020	5352	6661
4 years old	2547	4074	4668	3289	6207	3633	6305	3306	5734	3876	4331
5 years old	1361	1810	2491	2034	3693	2279	3664	2047	3459	2477	2486
6 years old	520	753	1124	948	1795	1402	1818	895	1921	1320	1223
**Gender**											
Male	2872	4296	4814	4910	7015	4793	7116	3965	8101	3414	5040
Female	2117	2980	3592	3603	5452	3610	5256	3098	6239	2481	3755
**Total**	2510	3657	4237	4293	6278	4235	6236	3554	7220	2961	4428

### Geographical distribution

**Fig. 2:**
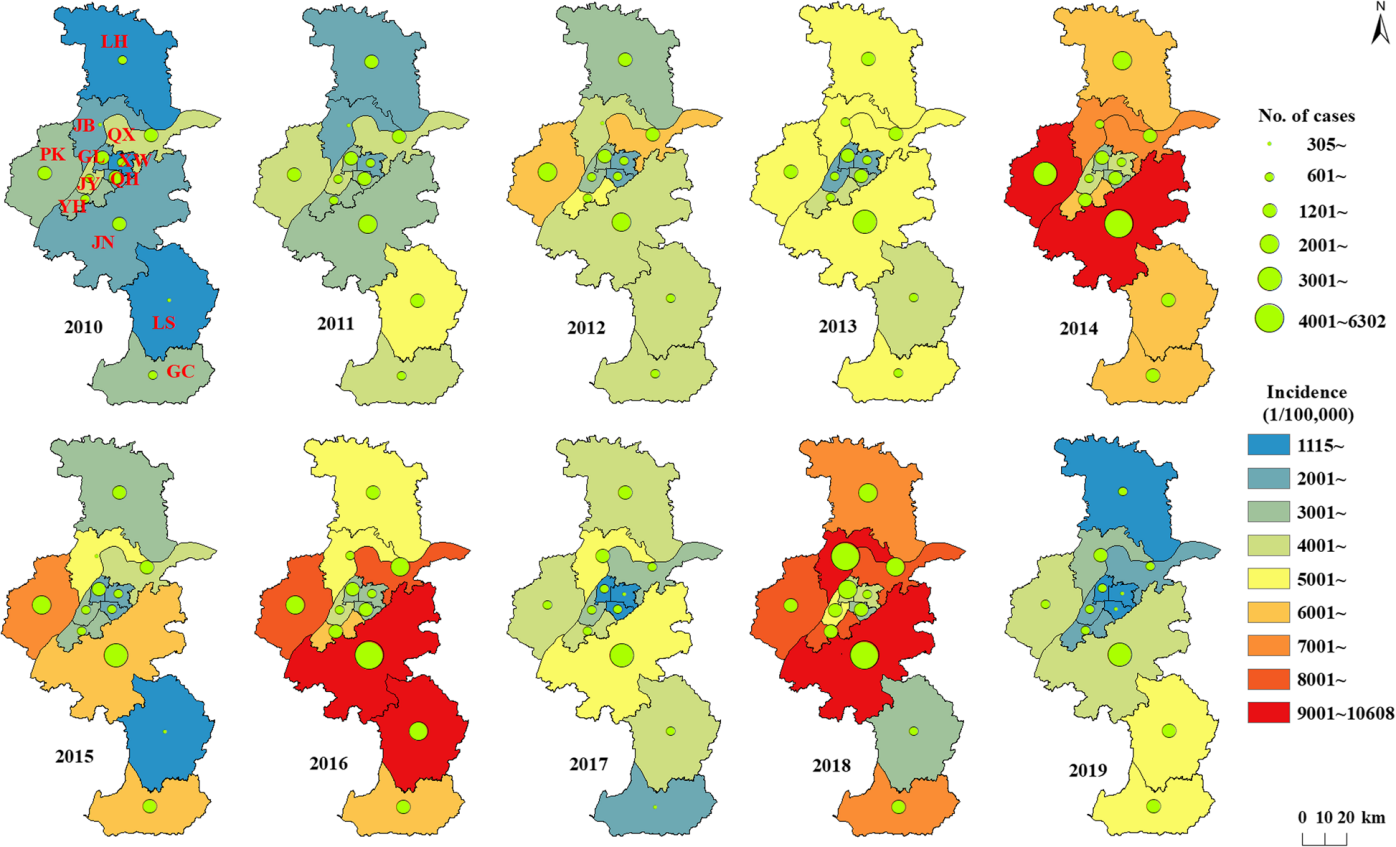
Geographic distribution of mild HFMD cases aged < 7 years in Nanjing, China, 2010–2019 (The size of circles represents the cumulative number of mild hand, foot and mouth disease (HFMD) cases aged < 7 years in each district in Nanjing, 2010–2019)

The top five districts in terms of the number of reported cases were Jiangning District (21.0%), Pukou District (10.5%), Liuhe District (9.1%), Gulou District (8.6%), and Qixia District (8.4%). The number of cases among these 5 districts accounted for 64.9% of the total number of cases. Jianning District showed the highest number of cases during 2010–2019. Jiangbei new Area had an increased number of cases in 2017–2019 (Fig. 2). Pukou District (6,338 cases/100,000) showed the highest average annual incidence of mild HFMD, followed by Jiangning District (5,953 cases/100,000).

### Spatial cluster

**Fig. 3:**
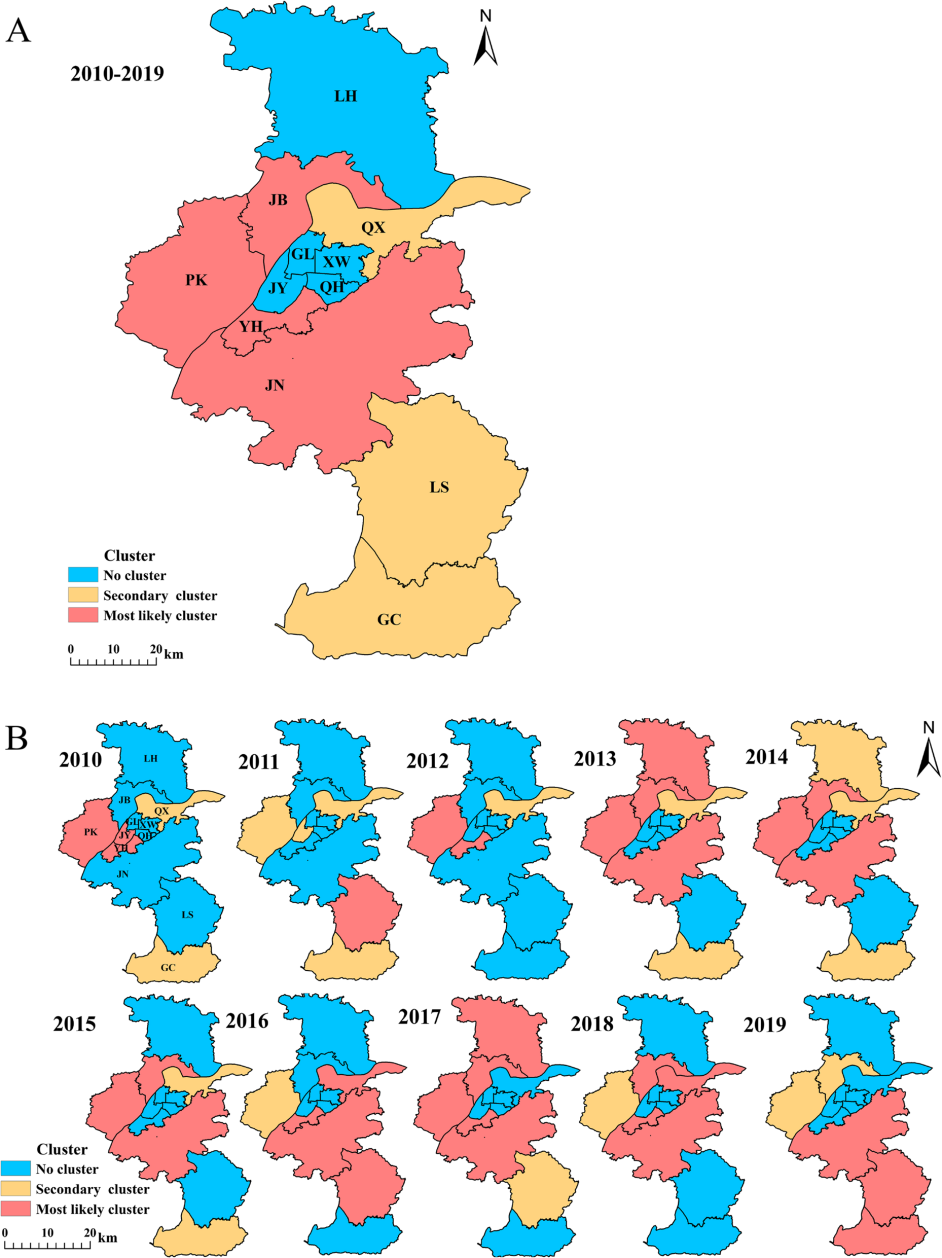
Spatial clusters of mild HFMD cases aged < 7 years in Nanjing, China, 2010–2019 (Panel (**A**) shows cumulative reported mild hand, foot and mouth disease (HFMD) cases aged < 7 years in each district in Nanjing, 2010–2019; Panel (**B**) shows yearly reported mild HFMD cases aged < 7 years in each district in Nanjing, 2010 to 2019)

For the cumulative number of mild cases aged < 7 years during 2010–2019, flexible spatial scan statistic detected an MLC (*LLR* = 3924.98, *P* < 0.001, overall *RR* = 1.30) consisting of 4 regions (Yuhuatai District, Jiangning District, Jiangbei new Area, and Pukou District). Flexible spatial scan statistic also detected two secondary clusters. For details, one secondary cluster included only one region (Qixia District) with *LLR* = 267.52, *P* < 0.001, and *RR* estimated at 1.21, while the other one included two regions (Lishui District and Gaochun District) with *LLR* = 155.78, *P* < 0.001, and the overall *RR* estimated of 1.12 (Fig. 3A and Supplementary Table 1).

The annual flexible spatial scan statistic showed that mild cases had spatial clusters each year (*P* < 0.05) from 2010–2019. The MLCs mostly included Jiangning District, Pukou District, Jiangbei new Area, and Yuhuatai District. The secondary clusters mostly included Qixia District and Gaochun District. Three regions (Xuanwu District, Qinhuai District, and Gulou District) showed no significant spatial cluster any year during 2010–2019. Jiangning District showed no significant spatial cluster between 2010 and 2012, while were included in the MLCs in the following years (Fig. 3B and Supplementary Table 1).

### Enterovirus serotype distribution

**Fig. 4:**
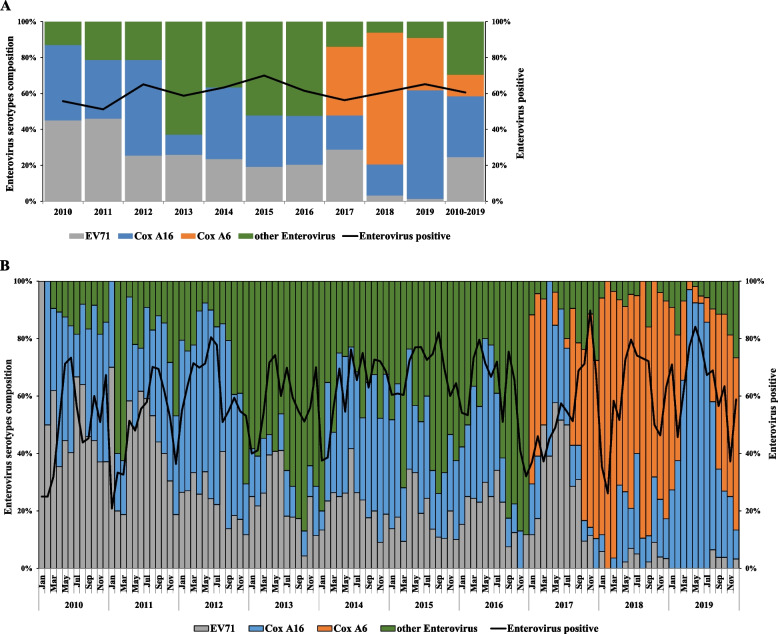
Enterovirus serotypes distribution for mild HFMD cases aged < 7 years in Nanjing, China, 2010–2019 (Continuous lines represent the rate of mild hand, foot, and mouth disease (HFMD) cases aged < 7 years tested positive for enterovirus in each year (**A**) and each month (**B**); the histogram shows the proportion of enterovirus serotypes among laboratory-confirmed mild HFMD cases aged < 7 years in each year (**A**) and each month (**B**). EV71: Enterovirus71, Cox A16: Coxsackievirus A16, Cox A6: Coxsackievirus A6. Other Enterovirus: other enterovirus positive test was defined as other non-EV71/Cox A16 enterovirus positive among 2010–2016, and other enterovirus positive test was defined as other non-EV71/Cox A16/Cox A6 enterovirus positive among 2017–2019)

Enterovirus tests were performed on 7,014 specimens from mild cases aged < 7 years. Among them, 4,249 specimens were tested with enterovirus positive (positive rate = 60.6%) (Supplementary Table 2). The annual positive rates of the enterovirus test ranged from 51.2 to 70.0% (Supplementary Table 2, Fig. 4A).

From 2010 to 2016, EV71, Cox A16, and other non-EV71/Cox A16 EVs, accounted for 29.1%, 34.6%, 36.3% of the cumulative enterovirus positive mild cases aged < 7 years, respectively. Notably, there was a significant difference in the annual distribution of enterovirus serotypes (χ^2^ = 528.36, *P* < 0.001). From 2017 to 2019, Cox A6, Cox A16, EV71, and other non-EV71/Cox A16/Cox A6 EVs, accounted for 47.3%, 32.5%, 10.7%, 9.5% of the cumulative enterovirus positive mild cases aged < 7 years, respectively. In addition, there was a significant difference in the annual distribution of enterovirus serotypes (χ^2^ = 375.72, *P* < 0.001) (Supplementary Table 2, Fig. 4A, and B).

As for the annual serotype distribution, EV71 was the dominant serotype both in 2010 (45.1%) and 2011 (46.0%) (Supplementary Table 2, Fig. 4A). However, the proportion of EV71 decreased since 2012, and was further reduced to less than 5.0% both in 2018 and 2019. Cox A16 became the dominant serotype in 2012, 2014, and 2019, accounting for 53.3%, 39.9%, 60.5%, respectively. Cox A6 was the dominant serotype both in 2017 (38.2%) and 2018 (73.4%). Other enteroviruses (non-EV71/Cox A16 EVs) made up a large proportion of the enterovirus positive mild cases between 2013 and 2016, accounting for 62.8%, 36.6%, 52.2%, 52.4%, respectively (Supplementary Table 2, Fig. 4B**)**.

## Discussion

Based on the data of 10-year surveillance of mild HFMD cases in Nanjing, our study firstly provided a systematic description of the epidemic trend of mild HFMD in Nanjing. Children aged < 7 years are a particularly vulnerable key age group, who had a much higher average annual incidence of mild HFMD than children aged ≥ 7 years. Moreover, mild cases aged < 7 years accounted for more than 90% of mild cases each year.

This study mainly focused on the epidemic trend of mild cases aged < 7 years. Several reasons may contribute to the susceptibility among children aged < 7 years, including the imperfect immune system, unhealthy hygiene habits (e.g. rarely hand washing, biting fingers), more exposure (e.g. touching toys, playing games), and a higher contact rate among kindergarten children [16]. In addition, due to the wide distribution and strong infectivity of enterovirus, most people have been infected with several local circulating enterovirus phenotypes in childhood, and most of them had developed immunity to HFMD as they get older [17, 18].

Consistent with other studies, the incidence of mild HFMD among children aged < 7 years was highest in children aged 1-year-old [19, 20], but lower both in children aged < 1 year and 6 years, the reason for which may be due to less contact with other infected children or protection from maternal antibodies [6, 19]. In addition, it may also be associated with older children entered into primary school, where provided comprehensive prevention and control measures, such as hand hygiene intervention, morning health check, case isolation, and disinfection. It is necessary to promote the development of good hygiene habits, especially hand hygiene in younger children. It is also important to raise awareness of the prevention and control of HFMD among parents, other guardians, and staffs in childcare facilities, especially the private nature of childcare facilities.

Males are more susceptible to mild HFMD than females, consistent with the findings reported in Shenzhen [21]. It may be due to the social factors that males have a wider range of activities and more exposure to infected people or contaminated items [22]. A systematic review [19] showed that males were significantly associated with a higher risk of HFMD (odds ratio range, 1.2–2.0) and more likely to develop symptoms, to be exposed to disease outbreaks, or to be taken by guardians to seek medical care.

Since 2014, the annual incidence of mild HFMD increased every other year and this trend had also been observed in other regions of China [23, 24], which may be related to the accumulation of susceptible populations and vaccination [25]. Mild cases occurred in semi-annual peaks of activity each year, consistent with the seasonal patterns of HFMD (including severe cases) reported in Chongqing [23], Xiamen [26], and other regions in southern China [9, 20, 27, 28]. In contrast, the regions of northern China mainly showed only one annual peak of HFMD, such as Shandong [29]. A study by Zhao et al*.* [30] showed that the seasonal patterns of HFMD were related to the interaction of school terms, spring festival, population flow, and meteorological factors. In this study, the seasonal patterns of mild cases in the pre-school children aged 0–3 years were the same as that of children aged < 7 years, which supports that school terms cannot fully explain the seasonal characteristics.

The peripheral urban areas were at higher risk of mild HFMD and showed a similar annular distribution. The seventh national census in Nanjing showed that, along with the successive introduction of the city’s regional development strategy, the population size of the main urban area has decreased, whereas the agglomeration effect of peripheral urban areas has caused its population size to expand [31]. The high population density and mobility in these peripheral urban areas might have contributed to the spread of HFMD, and bring difficulties to health education and vaccination. The rapid economic development of the peripheral urban areas has attracted many young couples to move in, hence the number of newborns has increased which in turn increased the size of the susceptible population, leading to an increased risk of HFMD infection. HFMD cases reported in Beijing were also mainly from peripheral urban areas [32]. A previous study reported that the high-risk areas of HFMD in China were mainly distributed in the districts, counties, or urban–rural areas surrounding provincial capitals, which might be related to population density, economic status, and cross-infection among the population [9]. For high risk areas, it is necessary to carry out active surveillance (e.g., enterovirus serotypes surveillance in the local population), special surveys (e.g., influence factors), inspections of childcare facilities on environmental hygiene, implementation of public health measures (e.g., morning health check), and diagnostic ability of local medical institutions to identify the weak points of prevention and control of mild HFMD timely.

The change in the distribution of enterovirus serotypes in different years may be related to the alternation of the predominant serotype, and the variation and recombination of the enterovirus with time [20]. In recent 10 years, Cox A16 was the dominant serotype among mild cases with enterovirus positive, while the dominant serotype in severe cases reported in Nanjing was EV71 [33]. With the implementation of the EV71 vaccination program, patients infected with EV71 have been reduced to some extent [20], thus the proportion of EV71 in the distribution of enterovirus serotypes has decreased.

Since the outbreak of HFMD caused by Cox A6 occurred in Finland in 2008, Cox A6 has become one of the dominant serotypes that could cause HFMD outbreaks globally after EV71 and Cox A16 [34–37]. Many areas in China also reported an increase of HFMD infected with Cox A6 in recent years, including Beijing [38], Fujian [39], Shenzhen [40], and Hangzhou [7]. Since 2017, Cox A6 has been added to the enterovirus serotypes surveillance in Nanjing, which became the predominant serotype in the following two years. It has been suggested that Cox A6 might account for a larger proportion of other non-EV71/Cox A16 EVs since 2013. Data at the national level also showed that mild cases infected with other non-EV71/Cox A16 EVs have been increasing, and of them 80% in 2013 and 59% in 2015 were infected with Cox A6 by further serotyping [41]. Apart from Cox A6, Cox A10 is also becoming another serotype with an increased prevalence [42, 43]. Therefore, it is necessary to increase the types of routine serotypes surveillance in the future to monitor the dynamic changes of enterovirus serotypes and develop monovalent or multivalent combined vaccines against other newly emerged dominant serotypes, such as Cox A16 and Cox A6.

Our study has several limitations that should be acknowledged when interpreting the results. First, this study only enrolled HFMD cases in the surveillance system. As most of enterovirus infections are sub-clinical and self-limited, patients may not seek medical care, which may lead to selection bias and information bias. Various measures have been continually implemented to control these biases, such as regular training on guidelines for the diagnosis and reporting of HFMD were provided to related health workers, supervision and assessment of the report quality, and strengthening the active surveillance. Second, the spatial scan was based on district level, limiting the collection of demography data at the street/town level. This may potentially miss some detailed information. In addition, Tango’s flexible spatial scan statistic can detect both circular and noncircular potential high-risk clusters, but it can only identify spatial clusters without time dimension at present [44, 45]. Lastly, the sample size of enterovirus serotypes surveillance was limited by the specimen collection. The ramp-up of specimen collection and laboratory testing capacity should be taken into account in the future.

## Conclusions

Children under 7 years old, especially 1-year-old, are a key age group with a higher risk of mild HFMD in Nanjing. In addition, males and people living in the areas surrounding central urban areas are at higher risk of mild HFMD. Future mitigation policies should take into account the high risk groups of the population and high risk areas. Cox A16 and Cox A6 became the dominant serotypes and they alternated or were co-epidemic. Involvement of more other enterovirus serotypes in the routine serotypes surveillance is highly recommended.

## Supplementary Information

**Additional file 1:** **Supplementary Table 1.** Spatial cluster scan of mild hand, foot, and mouth disease cases aged < 7 years in Nanjing, China, 2010-2019. **Supplementary Table 2.** Enterovirus serotypes distribution for mild hand, foot, and mouth disease cases aged < 7 years in Nanjing, China, 2010-2019.

**Additional file 2:** **Supplementary results.** Overview of epidemiological and etiological characteristics of mild hand, foot and mouth disease in children aged ≥ 7 years old, Nanjing, China, 2010‑2019.

## Data Availability

The data analysed during this study are included in this article. Some of the datasets are available from the corresponding author upon reasonable request.

## Abbreviations

HFMD: Hand, foot and mouth disease
DALYS: Disability-adjusted of life years
EV: Enterovirus
EV71: Enterovirus71
Cox A16: Coxsackievirus A16
Cox A6: Coxsackievirus A6
Cox A10: Coxsackievirus A10
CISDCP: The China Information System for Disease Control and Prevention
CDC: Center for Disease Control and Prevention
RT-PCR: Real-time reverse transcription-polymerase chain reaction
LLR: Restricted log likelihood ratio
MLC: Most likely cluster
RR: Relative risk

## References

[1] Zhu Q, Hao Y, Ma J, Yu S, Wang Y (2011). Surveillance of hand, foot, and mouth disease in mainland China (2008–2009). Biomed Environ Sci.

[2] Koh WM, Badaruddin H, La H, Chen MI, Cook AR (2018). Severity and burden of hand, foot and mouth disease in Asia: a modelling study. BMJ Glob Health.

[3] Notifiable Infectious Diseases Reports in China, 2019 (in Chinese). http://www.nhc.gov.cn/jkj/s3578/202004/b1519e1bc1a944fc8ec176db600f68d1.shtml. Accessed 29 June 2021.

[4] Gonzalez G, Carr MJ, Kobayashi M, Hanaoka N, Fujimoto T. Enterovirus-Associated Hand-Foot and Mouth Disease and Neurological Complications in Japan and the Rest of the World. Int J Mol Sci. 2019;20(20). 10.3390/ijms20205201. 10.3390/ijms20205201 PMC6834195 31635198

[5] The Picornavirus Pages: Enterovirus A. https://www.picornaviridae.com/sg3_ensavirinae/enterovirus/ev-a/ev-a.htm. Accessed 17 Dec 2021.

[6] Xing W, Liao Q, Viboud C, Zhang J, Sun J, Wu JT, Chang Z, Liu F, Fang VJ, Zheng Y (2014). Hand, foot, and mouth disease in China, 2008–12: an epidemiological study. Lancet Infect Dis.

[7] Wang J, Zhou J, Xie G, Zheng S, Lou B, Chen Y, Wu Y (2020). The Epidemiological and Clinical Characteristics of Hand, Foot, and Mouth Disease in Hangzhou, China, 2016 to 2018. Clin Pediatr (Phila).

[8] Zhou YJ, Niu XD, Ding YQ, Qian Z, Zhao BL (2021). Prevalence of recessive infection of pathogens of hand, foot, and mouth disease in healthy people in China: a meta-analysis. Medicine (Baltimore).

[9] Rui J, Luo K, Chen Q, Zhang D, Zhao Q, Zhang Y, Zhai X, Zhao Z, Zhang S, Liao Y (2021). Early warning of hand, foot, and mouth disease transmission: a modeling study in mainland, China. PLoS Negl Trop Dis.

[10] Li XW, Ni X, Qian SY, Wang Q, Jiang RM, Xu WB, Zhang YC, Yu GJ, Chen Q, Shang YX (2018). Chinese guidelines for the diagnosis and treatment of hand, foot and mouth disease (2018 edition). World J Pediatr.

[11] Ji H, Fan H, Lu P-X, Zhang X-F, Ai J, Shi C, Huo X, Bao C-J, Shan J, Jin Y (2019). Surveillance for severe hand, foot, and mouth disease from 2009 to 2015 in Jiangsu province: epidemiology, etiology, and disease burden. BMC Infect Dis.

[12] Tango T, Takahashi K (2005). A flexibly shaped spatial scan statistic for detecting clusters. Int J Health Geogr.

[13] Takahashi K, Yokoyama T, Tango T (2010). FleXScan v31: Software for the Flexible Scan Statistic Edited by Health NIoP.

[14] Tango T, Takahashi K (2012). A flexible spatial scan statistic with a restricted likelihood ratio for detecting disease clusters. Stat Med.

[15] Tango T (2008). A Spatial Scan Statistic with a Restricted Likelihood Ratio. Japan J Biom.

[16] Zhao J, Jiang F, Zhong L, Sun J, Ding J (2016). Age patterns and transmission characteristics of hand, foot and mouth disease in China. BMC Infect Dis.

[17] Wang J, Teng Z, Cui X, Li C, Pan H, Zheng Y, Mao S, Yang Y, Wu L, Guo X (2018). Epidemiological and serological surveillance of hand-foot-and-mouth disease in Shanghai, China, 2012–2016. Emerg Microbes Infect.

[18] Lanjuan L, Hong R (2013). Infectious Disease (in Chinese).

[19] Koh WM, Bogich T, Siegel K, Jin J, Chong EY, Tan CY, Chen MI, Horby P, Cook AR (2016). The epidemiology of hand, foot and mouth disease in Asia: a systematic review and analysis. Pediatr Infect Dis J.

[20] Chen B, Yang Y, Xu X, Zhao H, Li Y, Yin S, Chen YQ (2021). Epidemiological characteristics of hand, foot, and mouth disease in China: a meta-analysis. Medicine (Baltimore).

[21] Chen L, Yao XJ, Xu SJ, Yang H, Wu CL, Lu J, Xu WB, Zhang HL, Meng J, Zhang Y (2019). Molecular surveillance of coxsackievirus A16 reveals the emergence of a new clade in mainland China. Arch Virol.

[22] Chen GP, Wu JB, Wang JJ, Pan HF, Zhang J, Shi YL, Cao C, Li FR, Fan YG, Meng FY (2016). Epidemiological characteristics and influential factors of hand, foot and mouth disease (HFMD) reinfection in children in Anhui province. Epidemiol Infect.

[23] Qi L, Tang W, Zhao H, Ling H, Su K, Zhao H, Li Q, Shen T. Epidemiological Characteristics and Spatial-Temporal Distribution of Hand, Foot, and Mouth Disease in Chongqing, China, 2009–2016. Int J Environ Res Public Health. 2018;15(2). 10.3390/ijerph15020270. 10.3390/ijerph15020270 PMC5858339 29401726

[24] Xiao X, Liao Q, Kenward MG, Zheng Y, Huang J, Yin F, Yu H, Li X (2016). Comparisons between mild and severe cases of hand, foot and mouth disease in temporal trends: a comparative time series study from mainland China. BMC Public Health.

[25] Yang D, Liu R, Ye L, Hu Q, Rui J, Zhou Y, Zhang H, Zhang X, Zhao B, Chen T (2020). Hand, foot, and mouth disease in Changsha City, China, 2009–2017: a new method to analyse the epidemiological characteristics of the disease. Infect Dis (Lond).

[26] Huang Z, Wang M, Qiu L, Wang N, Zhao Z, Rui J, Wang Y, Liu X, Hannah MN, Zhao B (2019). Seasonality of the transmissibility of hand, foot and mouth disease: a modelling study in Xiamen City. China Epidemiol Infect.

[27] Liu W, Ji H, Shan J, Bao J, Sun Y, Li J, Bao C, Tang F, Yang K, Bergquist R (2015). Spatiotemporal dynamics of hand-foot-mouth disease and its relationship with meteorological factors in Jiangsu Province, China. PLoS One.

[28] Zhu J, Shi P, Zhou W, Chen X, Zhang X, Huang C, Zhang Q, Zhu X, Xu Q, Gao Y (2020). Assessment of temperature-hand, foot, and mouth disease association and its variability across urban and rural populations in Wuxi, China: a distributed lag nonlinear analysis. Am J Trop Med Hyg.

[29] Wang J, Hu T, Sun D, Ding S, Carr MJ, Xing W, Li S, Wang X, Shi W (2017). Epidemiological characteristics of hand, foot, and mouth disease in Shandong, China, 2009–2016. Sci Rep.

[30] Zhao J, Hu X (2019). The complex transmission seasonality of hand, foot, and mouth disease and its driving factors. BMC Infect Dis.

[31] Grasp the population changes in the new era from a comprehensive perspective: interpretation of the seventh national census in Nanjing (in Chinese). http://tjj.nanjing.gov.cn/njstjj/202105/t20210524_2945811.html. Accessed 10 Nov 2021.

[32] Tian L, Liang F, Xu M, Jia L, Pan X, Clements ACA (2018). Spatio-temporal analysis of the relationship between meteorological factors and hand-foot-mouth disease in Beijing, China. BMC Infect Dis.

[33] Ma T, Xie G, Sun H, Xu Q, Feng L, Xu Y, Sui H, Lin D, Zhang M (2018). Epidemiological characteristics and temporal-spatial clustering analysis of severe hand-foot-mouth disease in Nanjing from 2009 to 2016 (in Chinese). Chin J Dis Control Prev.

[34] Bian L, Wang Y, Yao X, Mao Q, Xu M, Liang Z (2015). Coxsackievirus A6: a new emerging pathogen causing hand, foot and mouth disease outbreaks worldwide. Expert Rev Anti Infect Ther.

[35] Wu Y, Yeo A, Phoon MC, Tan EL, Poh CL, Quak SH, Chow VT (2010). The largest outbreak of hand; foot and mouth disease in Singapore in 2008: the role of enterovirus 71 and coxsackievirus A strains. Int J Infect Dis.

[36] Fujimoto T, Iizuka S, Enomoto M, Abe K, Yamashita K, Hanaoka N, Okabe N, Yoshida H, Yasui Y, Kobayashi M (2012). Hand, foot, and mouth disease caused by coxsackievirus A6, Japan, 2011. Emerg Infect Dis.

[37] Puenpa J, Chieochansin T, Linsuwanon P, Korkong S, Thongkomplew S, Vichaiwattana P, Theamboonlers A, Poovorawan Y (2013). Hand, foot, and mouth disease caused by coxsackievirus A6, Thailand, 2012. Emerg Infect Dis.

[38] Hongyan G, Chengjie M, Qiaozhi Y, Wenhao H, Juan L, Lin P, Yanli X, Hongshan W, Xingwang L (2014). Hand, foot and mouth disease caused by coxsackievirus A6, Beijing, 2013. Pediatr Infect Dis J.

[39] Chen W, Weng YW, He WX, Zhang YJ, Yang XH, Meng H, Xie JF, Wang JZ, Zheng KC, Yan YS (2014). Molecular epidemiology of HFMD-associated pathogen coxsackievirus A6 in Fujian Province, 2011-2013 (in Chinese). Chin J Virology.

[40] Chen L, Xu SJ, Yao XJ, Yang H, Zhang HL, Meng J, Zeng HR, Huang XH, Zhang RL, He YQ (2020). Molecular epidemiology of enteroviruses associated with severe hand, foot and mouth disease in Shenzhen, China, 2014–2018. Arch Virol.

[41] Li Y, Chang Z, Wu P, Liao Q, Liu F, Zheng Y, Luo L, Zhou Y, Chen Q, Yu S (2018). Emerging Enteroviruses Causing Hand, Foot and Mouth Disease, China, 2010–2016. Emerg Infect Dis.

[42] Schildgen O, Tian H, Zhang Y, Shi Y, Li X, Sun Q, Liu L, Zhao D, Xu B. Epidemiological and aetiological characteristics of hand, foot, and mouth disease in Shijiazhuang City, Hebei province, China, 2009–2012. Plos One 2017;12(5). 10.1371/journal.pone.0176604. 10.1371/journal.pone.0176604 PMC5423607 28486500

[43] Fu X, Wan Z, Li Y, Hu Y, Jin X, Zhang C (2020). National epidemiology and evolutionary history of four hand, foot and mouth disease-related enteroviruses in China from 2008 to 2016. Virologica Sinica.

[44] Zhou J, Feng Z, Tang K, Li X (2010). Study on the application of flexible spatial scan statistic to spatial aggregation of infection disease (in Chinese). Chin J Dis Control Prev.

[45] FleXScan User Guide for version 3.1. http://www.niph.go.jp/soshiki/gijutsu/index_e.html Accessed 10 June 2021.

