# Supplementary material for: Epidemiological and etiological characteristics of mild hand, foot and mouth disease in children under 7 years old, Nanjing, China, 2010–2019
Source: Arch Public Health. 2022 Oct 8;80:220. doi: 10.1186/s13690-022-00974-4 (PMC9548167; doi:10.1186/s13690-022-00974-4)
Supplement: Supplementary file 1 — Additional file 1: Supplementary Table 1. Spatial cluster scan of mild hand, foot, and mouth disease cases aged < 7 years in Nanjing, China, 2010-2019. Supplementary Table 2. Enterovirus serotypes distribution for mild hand, foot, and mouth disease cases aged < 7 years in Nanjing, China, 2010-2019. [file 13690_2022_974_MOESM1_ESM.docx]

**Additional file 1**

**Supplementary Table 1.** Spatial cluster scan of mild hand, foot, and mouth disease cases aged < 7 years in Nanjing, China, 2010-2019

| **Year** | **Clusters** | **Districts in clusters** | **Observed number of cases** | **Expected number of cases** | **LLR^*^** | **RR** | **P-value** |
| --- | --- | --- | --- | --- | --- | --- | --- |
| 2010-2019 | Most likely cluster | YH,JN,JB,PK | 77053 | 59155.00 | 3924.98 | 1.30 | <0.001 |
|  | Secondary cluster1 | QX | 14694 | 12158.40 | 267.52 | 1.21 | <0.001 |
|  | Secondary cluster2 | LS,GC | 23409 | 20971.70 | 155.78 | 1.12 | <0.001 |
| 2010 | Most likely cluster | YH,JY,PK | 3039 | 2143.42 | 207.19 | 1.42 | <0.001 |
|  | Secondary cluster1 | QX | 1357 | 900.60 | 109.41 | 1.51 | <0.001 |
|  | Secondary cluster2 | GC | 832 | 725.48 | 7.97 | 1.15 | 0.008 |
| 2011 | Most likely cluster | LS | 1343 | 856.40 | 126.22 | 1.57 | <0.001 |
|  | Secondary cluster1 | JY,PK | 2415 | 1942.92 | 61.96 | 1.24 | <0.001 |
|  | Secondary cluster2 | QX | 1219 | 1051.50 | 13.71 | 1.16 | <0.001 |
|  | Secondary cluster3 | GC | 987 | 846.40 | 11.79 | 1.17 | <0.001 |
| 2012 | Most likely cluster | YH,PK | 3206 | 2344.87 | 169.25 | 1.37 | <0.001 |
|  | Secondary cluster | QX | 1564 | 1053.39 | 116.31 | 1.48 | <0.001 |
| 2013 | Most likely cluster | JN,LH,JB,PK | 7294 | 5808.84 | 292.24 | 1.26 | <0.001 |
|  | Secondary cluster1 | QX | 1380 | 1072.34 | 43.68 | 1.29 | <0.001 |
|  | Secondary cluster2 | GC | 1040 | 883.28 | 13.98 | 1.18 | <0.001 |
| 2014 | Most likely cluster | JN,JB,PK | 9297 | 6356.63 | 864.97 | 1.46 | <0.001 |
|  | Secondary cluster1 | QX,LH | 4204 | 3792.98 | 25.86 | 1.11 | <0.001 |
|  | Secondary cluster2 | GC | 1488 | 1347.35 | 7.55 | 1.10 | 0.015 |
| 2015 | Most likely cluster | JN,JB,PK | 6430 | 4298.63 | 669.10 | 1.50 | <0.001 |
|  | Secondary cluster1 | GC | 1433 | 911.35 | 136.18 | 1.57 | <0.001 |
|  | Secondary cluster2 | QX | 1255 | 1069.41 | 16.42 | 1.17 | <0.001 |
| 2016 | Most likely cluster | LS,YH,JN,QX | 11475 | 7834.02 | 1206.40 | 1.46 | <0.001 |
|  | Secondary cluster | PK | 2896 | 2093.54 | 152.61 | 1.38 | <0.001 |
| 2017 | Most likely cluster | YH,JN,LH,JB,PK | 7786 | 5483.53 | 823.24 | 1.42 | <0.001 |
|  | Secondary cluster | LS | 1080 | 788.36 | 51.81 | 1.37 | <0.001 |
| 2018 | Most likely cluster | YH,JN,QX,JB | 14638 | 10550.30 | 1243.93 | 1.39 | <0.001 |
|  | Secondary cluster | PK | 1687 | 1470.32 | 16.14 | 1.15 | <0.001 |
| 2019 | Most likely cluster | LS,GC,JN | 6587 | 4121.10 | 971.09 | 1.60 | <0.001 |
|  | Secondary cluster | JB,PK | 2272 | 1782.91 | 71.83 | 1.27 | <0.001 |

**^*^**LLR, restriction log likelihood ratio along with the value of parameter alpha set as 0.2; YH, Yuhuatai District; JN, Jiangning District; QX, Qixia new District; JB, Jiangbei new Area; PK, Pukou District; LS, Lishui District; GC, Gaochun District; JY, Jianye District; QH, Qinhuai District; LH, Liuhe District.

**Supplementary Table2.** Enterovirus serotypes distribution for mild hand, foot, and mouth disease cases aged < 7 years in Nanjing, China, 2010-2019

| **Year** | **Number of specimens** | **Enterovirus positive (n, %)** | **Enterovirus serotypes (n, %)** | | | | |
| --- | --- | --- | --- | --- | --- | --- | --- |
|  |  |  | **EV71 (n, %)** | **Cox A16 (n, %)** | **Cox A6 (n, %)** | **Other enterovirus^*^ (n, %)** | |
| 2010 | 745 | 415 (55.7) | 187 (45.1) | 174 (41.9) | — | 54 (13.0) |  |
| 2011 | 908 | 465 (51.2) | 214 (46.0) | 152 (32.7) | — | 99 (21.3) |  |
| 2012 | 872 | 568 (65.1) | 144 (25.4) | 303 (53.3) | — | 121 (21.3) |  |
| 2013 | 682 | 401 (58.8) | 104 (25.9) | 45 (11.2) | — | 252 (62.9) |  |
| 2014 | 699 | 443 (63.4) | 104 (23.5) | 177 (39.9) | — | 162 (36.6) |  |
| 2015 | 720 | 504 (70.0) | 97 (19.2) | 144 (28.6) | — | 263 (52.2) |  |
| 2016 | 639 | 393 (61.5) | 80 (20.4) | 107 (27.2) | — | 206 (52.4) |  |
| 2017 | 595 | 335 (56.3) | 96 (28.7) | 64 (19.1) | 128 (38.2) | 47 (14.0) |  |
| 2018 | 606 | 368 (60.7) | 12 (3.2) | 64 (17.4) | 270 (73.4) | 22 (6.0) |  |
| 2019 | 548 | 357 (65.1) | 5 (1.4) | 216 (60.5) | 104 (29.1) | 32 (9.0) |  |
| Total | 7014 | 4249 (60.6) | 1043 (24.5) | 1446 (34.0) | 502 (11.8) | 1258 (29.6) |  |

**EV71**: Enterovirus 71, **Cox A16**: Coxsackievirus A16, **Cox A6**: Coxsackievirus A6.

^*^**Other enterovirus:** Since Cox A6 serotype was not added to enterovirus serotype surveillance until 2017, other enterovirus positive test was defined as other non-EV71/Cox A16 enteroviruses positive among 2010-2016, and other enterovirus positive test was defined as other non-EV71/Cox A16/Cox A6 enteroviruses positive among 2017-2019.
