# Supplementary material for: Epidemiological and etiological characteristics of mild hand, foot and mouth disease in children under 7 years old, Nanjing, China, 2010–2019
Source: Arch Public Health. 2022 Oct 8;80:220. doi: 10.1186/s13690-022-00974-4 (PMC9548167; doi:10.1186/s13690-022-00974-4)
Supplement: Supplementary file 2 — Additional file 2: Supplementary results. Overview of epidemiological and etiological characteristics of mild hand, foot and mouth disease in children aged ≥ 7 years old, Nanjing, China, 2010‑2019. [file 13690_2022_974_MOESM2_ESM.doc]

**Supplementary results**

**Overview of epidemiological and etiological characteristics of mild hand, foot and mouth disease in children aged ≥ 7 years old, Nanjing, China, 2010‑2019**

A total of 10,378 mild HFMD cases (hereinafter “mild cases”) aged ≥ 7 years old, accounting for 5.6% of all mild cases, were reported to the system of the China Information System for Disease Control and Prevention (CISDCP) in Nanjing during 2010-2019. The 10 years (2010-2019) average annual incidence of mild cases aged ≥ 7 years old was 14 cases/100,000 (range from 7 cases/100,000 to 28 cases/100,000), which was much lower than that of mild cases aged < 7 years with the average annual incidence of 4,428 cases/100,000 (range from 2,510 cases/100,000 to 7,220 cases/100,000) (*P* < 0.001). The annual incidence of mild cases aged ≥ 7 years increased every other year since 2014, which is similar to mild cases aged < 7 years old. As for the seasonal characteristics, mild cases showed a predominant peak (April to July) and a smaller peak (October to December), which accounting for 58.05% (6,024/10378) and 21.94% (2,277/10378) of the mild cases aged ≥ 7 years old over the past 10 years. Mild cases among children aged ≥ 7 years old mostly occurred in adolescents and very occasionally in adults, and the median age was 8 years old (range 7 years old to 89 years old). The average annual incidence of males (15 cases/100,000) was significantly higher than females (12 cases/100,000) (*χ^2^*=128.33, *P* < 0.001).

Over the past decade, the whole number of mild cases aged ≥ 7 in Jiangning District ranked first in Nanjing, followed by Gulou District. The average annual incidence of mild cases aged ≥ 7 in Jiangbei new Area (26 cases/100,000) ranked first in Nanjing, followed by Jiangning District (18 cases/100,000).

The surveillance of HFMD including the enterovirus (EV) serotypes surveillance. Coxsackievirus A6 (Cox A6) serotype was added to the routine enterovirus serotypes surveillance since 2017. A total of 444 specimens from mild cases aged ≥ 7 years were tested for enterovirus. Of them, 284 specimens tested enterovirus positive (positive rate=64.0%). During 2010-2016, EV71, Cox A16, and other non-EV71/Cox A16 EVs, accounted for 27.7%, 32.8%, 39.5% of all the enterovirus test positive cases aged ≥ 7 years, respectively. Notably, there was a significant difference in the annual distribution of enterovirus serotypes (χ^2^=51.68, *P* < 0.001). During 2017-2019, Cox A6, Cox A16, EV71, and other non-EV71/Cox A16/Cox A6 EVs, accounted for 43.0%, 34.6%, 13.1%, 9.3% of the enterovirus positive mild cases aged ≥ 7 years, respectively. In addition, there was a significant difference in the annual distribution of enterovirus serotypes (χ^2^=27.94, *P* < 0.001). The trend of etiologic change in mild cases aged ≥ 7 years was similar to that in mild cases younger than 7 years old.
